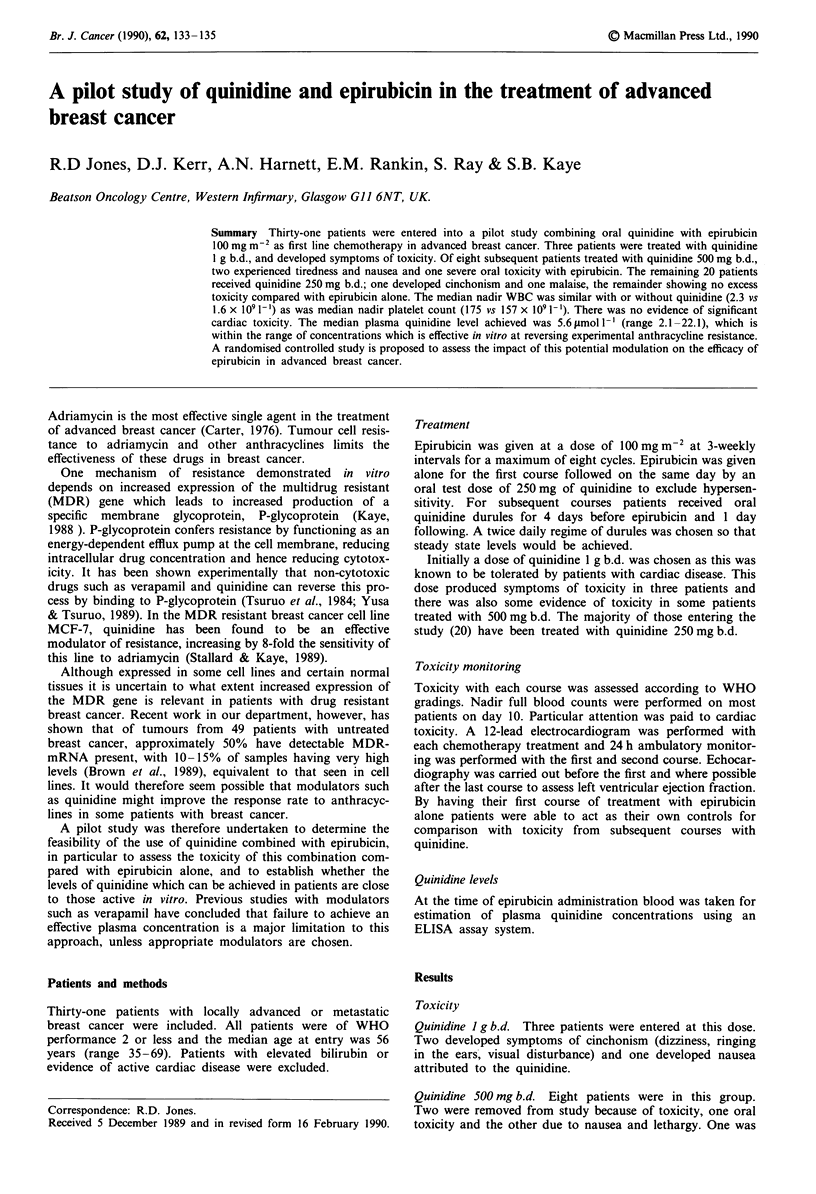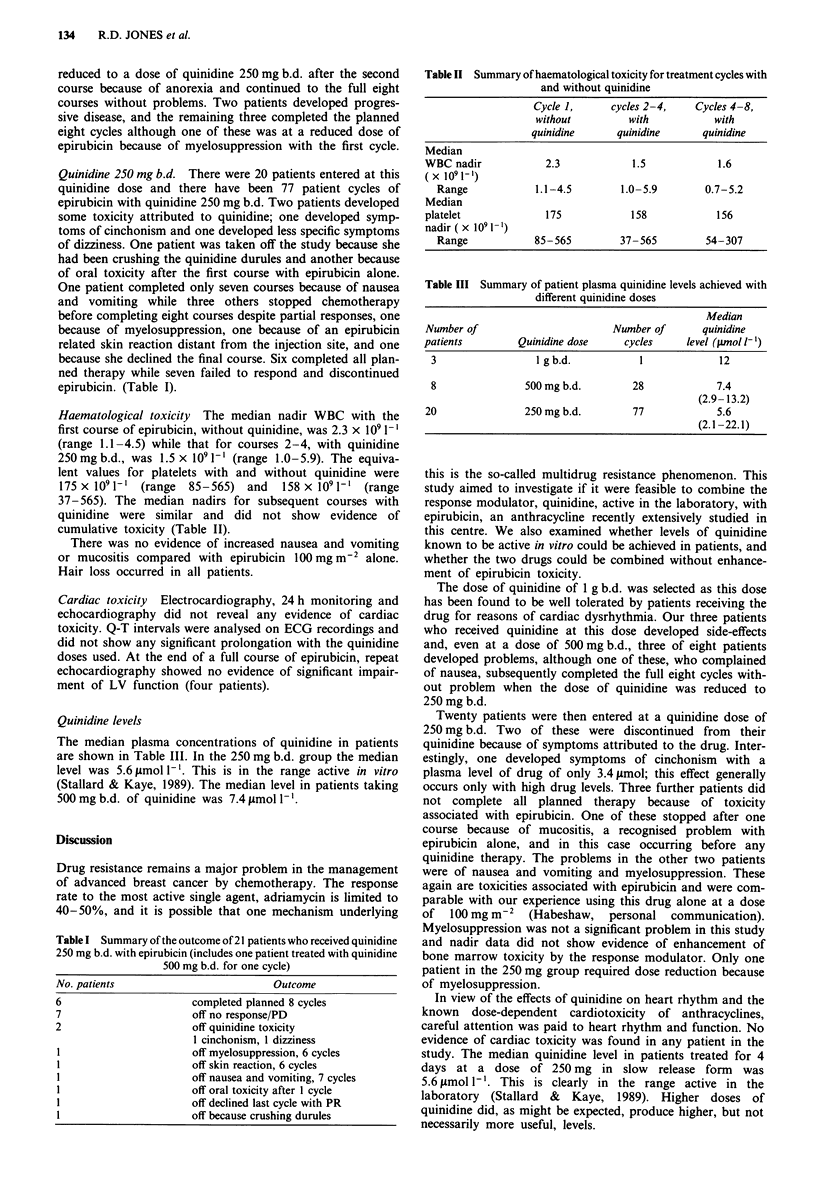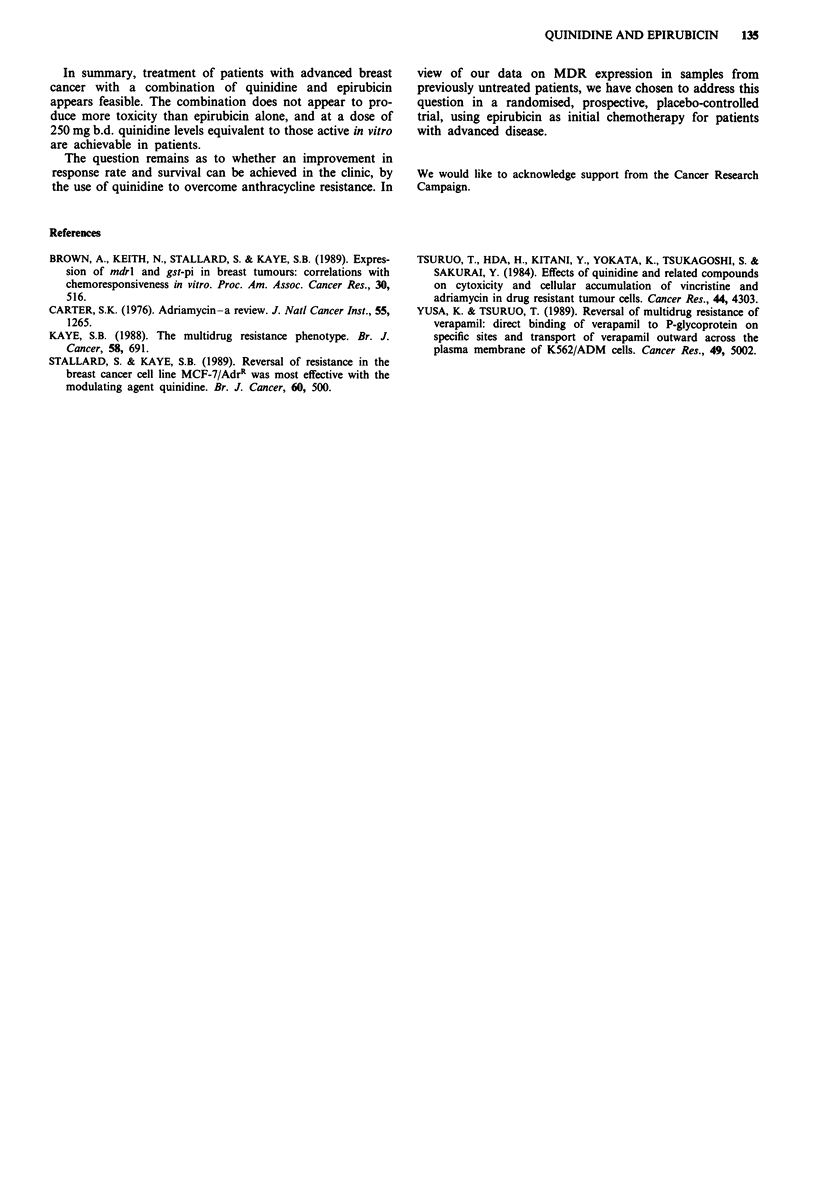# A pilot study of quinidine and epirubicin in the treatment of advanced breast cancer.

**DOI:** 10.1038/bjc.1990.244

**Published:** 1990-07

**Authors:** R. D. Jones, D. J. Kerr, A. N. Harnett, E. M. Rankin, S. Ray, S. B. Kaye

**Affiliations:** Beatson Oncology Centre, Western Infirmary, Glasgow, UK.

## Abstract

Thirty-one patients were entered into a pilot study combining oral quinidine with epirubicin 100 mg m-2 as first line chemotherapy in advanced breast cancer. Three patients were treated with quinidine 1 g b.d., and developed symptoms of toxicity. Of eight subsequent patients treated with quinidine 500 mg b.d., two experienced tiredness and nausea and one severe oral toxicity with epirubicin. The remaining 20 patients received quinidine 250 mg b.d.; one developed cinchonism and one malaise, the remainder showing no excess toxicity compared with epirubicin alone. The median nadir WBC was similar with or without quinidine (2.3 vs 1.6 x 10(9) l-1) as was median nadir platelet count (175 vs 157 x 10(9) l-1). There was no evidence of significant cardiac toxicity. The median plasma quinidine level achieved was 5.6 mumol l-1 (range 2.1-22.1), which is within the range of concentrations which is effective in vitro at reversing experimental anthracycline resistance. A randomised controlled study is proposed to assess the impact of this potential modulation on the efficacy of epirubicin in advanced breast cancer.


Br. J. Cancer (1990), 62, 133-135 © Macmillan Press Ltd., 1990

### A pilot study of quinidine and epirubicin in the treatment of advanced breast cancer

R.D Jones, D.J. Kerr, A.N. Harnett, E.M. Rankin, S. Ray & S.B. Kaye

*Beatson Oncology Centre, Western Infirmary, Glasgow G11 6NT, UK.*

**Summary** Thirty-one patients were entered into a pilot study combining oral quinidine with epirubicin 100 mg $m^{-2}$ as first line chemotherapy in advanced breast cancer. Three patients were treated with quinidine 1 g b.d., and developed symptoms of toxicity. Of eight subsequent patients treated with quinidine 500 mg b.d., two experienced tiredness and nausea and one severe oral toxicity with epirubicin. The remaining 20 patients received quinidine 250 mg b.d.; one developed cinchonism and one malaise, the remainder showing no excess toxicity compared with epirubicin alone. The median nadir WBC was similar with or without quinidine (2.3 *vs* $1.6 \times 10^9 l^{-1}$) as was median nadir platelet count (175 *vs* $157 \times 10^9 l^{-1}$). There was no evidence of significant cardiac toxicity. The median plasma quinidine level achieved was 5.6 μmol $l^{-1}$ (range 2.1-22.1), which is within the range of concentrations which is effective *in vitro* at reversing experimental anthracycline resistance. A randomised controlled study is proposed to assess the impact of this potential modulation on the efficacy of epirubicin in advanced breast cancer.

Adriamycin is the most effective single agent in the treatment of advanced breast cancer (Carter, 1976). Tumour cell resistance to adriamycin and other anthracyclines limits the effectiveness of these drugs in breast cancer.

One mechanism of resistance demonstrated *in vitro* depends on increased expression of the multidrug resistant (MDR) gene which leads to increased production of a specific membrane glycoprotein, P-glycoprotein (Kaye, 1988 ). P-glycoprotein confers resistance by functioning as an energy-dependent efflux pump at the cell membrane, reducing intracellular drug concentration and hence reducing cytotoxicity. It has been shown experimentally that non-cytotoxic drugs such as verapamil and quinidine can reverse this process by binding to P-glycoprotein (Tsuruo *et al.*, 1984; Yusa & Tsuruo, 1989). In the MDR resistant breast cancer cell line MCF-7, quinidine has been found to be an effective modulator of resistance, increasing by 8-fold the sensitivity of this line to adriamycin (Stallard & Kaye, 1989).

Although expressed in some cell lines and certain normal tissues it is uncertain to what extent increased expression of the MDR gene is relevant in patients with drug resistant breast cancer. Recent work in our department, however, has shown that of tumours from 49 patients with untreated breast cancer, approximately 50% have detectable MDR-mRNA present, with 10-15% of samples having very high levels (Brown *et al.*, 1989), equivalent to that seen in cell lines. It would therefore seem possible that modulators such as quinidine might improve the response rate to anthracyclines in some patients with breast cancer.

A pilot study was therefore undertaken to determine the feasibility of the use of quinidine combined with epirubicin, in particular to assess the toxicity of this combination compared with epirubicin alone, and to establish whether the levels of quinidine which can be achieved in patients are close to those active *in vitro*. Previous studies with modulators such as verapamil have concluded that failure to achieve an effective plasma concentration is a major limitation to this approach, unless appropriate modulators are chosen.

### Patients and methods

Thirty-one patients with locally advanced or metastatic breast cancer were included. All patients were of WHO performance 2 or less and the median age at entry was 56 years (range 35-69). Patients with elevated bilirubin or evidence of active cardiac disease were excluded.

Correspondence: R.D. Jones.
Received 5 December 1989 and in revised form 16 February 1990.

### *Treatment*

Epirubicin was given at a dose of 100 mg $m^{-2}$ at 3-weekly intervals for a maximum of eight cycles. Epirubicin was given alone for the first course followed on the same day by an oral test dose of 250 mg of quinidine to exclude hypersensitivity. For subsequent courses patients received oral quinidine durules for 4 days before epirubicin and 1 day following. A twice daily regime of durules was chosen so that steady state levels would be achieved.

Initially a dose of quinidine 1 g b.d. was chosen as this was known to be tolerated by patients with cardiac disease. This dose produced symptoms of toxicity in three patients and there was also some evidence of toxicity in some patients treated with 500 mg b.d. The majority of those entering the study (20) have been treated with quinidine 250 mg b.d.

### *Toxicity monitoring*

Toxicity with each course was assessed according to WHO gradings. Nadir full blood counts were performed on most patients on day 10. Particular attention was paid to cardiac toxicity. A 12-lead electrocardiogram was performed with each chemotherapy treatment and 24 h ambulatory monitoring was performed with the first and second course. Echocardiography was carried out before the first and where possible after the last course to assess left ventricular ejection fraction. By having their first course of treatment with epirubicin alone patients were able to act as their own controls for comparison with toxicity from subsequent courses with quinidine.

### *Quinidine levels*

At the time of epirubicin administration blood was taken for estimation of plasma quinidine concentrations using an ELISA assay system.

### Results

### *Toxicity*

*Quinidine 1 g b.d.* Three patients were entered at this dose. Two developed symptoms of cinchonism (dizziness, ringing in the ears, visual disturbance) and one developed nausea attributed to the quinidine.

*Quinidine 500 mg b.d.* Eight patients were in this group. Two were removed from study because of toxicity, one oral toxicity and the other due to nausea and lethargy. One was reduced to a dose of quinidine 250 mg b.d. after the second course because of anorexia and continued to the full eight courses without problems. Two patients developed progressive disease, and the remaining three completed the planned eight cycles although one of these was at a reduced dose of epirubicin because of myelosuppression with the first cycle.

*Quinidine 250 mg b.d.* There were 20 patients entered at this quinidine dose and there have been 77 patient cycles of epirubicin with quinidine 250 mg b.d. Two patients developed some toxicity attributed to quinidine; one developed symptoms of cinchonism and one developed less specific symptoms of dizziness. One patient was taken off the study because she had been crushing the quinidine durules and another because of oral toxicity after the first course with epirubicin alone. One patient completed only seven courses because of nausea and vomiting while three others stopped chemotherapy before completing eight courses despite partial responses, one because of myelosuppression, one because of an epirubicin related skin reaction distant from the injection site, and one because she declined the final course. Six completed all planned therapy while seven failed to respond and discontinued epirubicin. (Table I).

*Haematological toxicity* The median nadir WBC with the first course of epirubicin, without quinidine, was $2.3 \times 10^9\ l^{-1}$ (range 1.1–4.5) while that for courses 2–4, with quinidine 250 mg b.d., was $1.5 \times 10^9\ l^{-1}$ (range 1.0–5.9). The equivalent values for platelets with and without quinidine were $175 \times 10^9\ l^{-1}$ (range 85–565) and $158 \times 10^9\ l^{-1}$ (range 37–565). The median nadirs for subsequent courses with quinidine were similar and did not show evidence of cumulative toxicity (Table II).

There was no evidence of increased nausea and vomiting or mucositis compared with epirubicin 100 mg $m^{-2}$ alone. Hair loss occurred in all patients.

*Cardiac toxicity* Electrocardiography, 24 h monitoring and echocardiography did not reveal any evidence of cardiac toxicity. Q-T intervals were analysed on ECG recordings and did not show any significant prolongation with the quinidine doses used. At the end of a full course of epirubicin, repeat echocardiography showed no evidence of significant impairment of LV function (four patients).

### *Quinidine levels*

The median plasma concentrations of quinidine in patients are shown in Table III. In the 250 mg b.d. group the median level was 5.6 $\mu mol\ l^{-1}$. This is in the range active *in vitro* (Stallard & Kaye, 1989). The median level in patients taking 500 mg b.d. of quinidine was 7.4 $\mu mol\ l^{-1}$.

### Discussion

Drug resistance remains a major problem in the management of advanced breast cancer by chemotherapy. The response rate to the most active single agent, adriamycin is limited to 40–50%, and it is possible that one mechanism underlying this is the so-called multidrug resistance phenomenon. This study aimed to investigate if it were feasible to combine the response modulator, quinidine, active in the laboratory, with epirubicin, an anthracycline recently extensively studied in this centre. We also examined whether levels of quinidine known to be active *in vitro* could be achieved in patients, and whether the two drugs could be combined without enhancement of epirubicin toxicity.

The dose of quinidine of 1 g b.d. was selected as this dose has been found to be well tolerated by patients receiving the drug for reasons of cardiac dysrhythmia. Our three patients who received quinidine at this dose developed side-effects and, even at a dose of 500 mg b.d., three of eight patients developed problems, although one of these, who complained of nausea, subsequently completed the full eight cycles without problem when the dose of quinidine was reduced to 250 mg b.d.

Twenty patients were then entered at a quinidine dose of 250 mg b.d. Two of these were discontinued from their quinidine because of symptoms attributed to the drug. Interestingly, one developed symptoms of cinchonism with a plasma level of drug of only 3.4 μmol; this effect generally occurs only with high drug levels. Three further patients did not complete all planned therapy because of toxicity associated with epirubicin. One of these stopped after one course because of mucositis, a recognised problem with epirubicin alone, and in this case occurring before any quinidine therapy. The problems in the other two patients were of nausea and vomiting and myelosuppression. These again are toxicities associated with epirubicin and were comparable with our experience using this drug alone at a dose of 100 mg $m^{-2}$ (Habeshaw, personal communication). Myelosuppression was not a significant problem in this study and nadir data did not show evidence of enhancement of bone marrow toxicity by the response modulator. Only one patient in the 250 mg group required dose reduction because of myelosuppression.

In view of the effects of quinidine on heart rhythm and the known dose-dependent cardiotoxicity of anthracyclines, careful attention was paid to heart rhythm and function. No evidence of cardiac toxicity was found in any patient in the study. The median quinidine level in patients treated for 4 days at a dose of 250 mg in slow release form was 5.6 $\mu mol\ l^{-1}$. This is clearly in the range active in the laboratory (Stallard & Kaye, 1989). Higher doses of quinidine did, as might be expected, produce higher, but not necessarily more useful, levels.

**Table I** Summary of the outcome of 21 patients who received quinidine 250 mg b.d. with epirubicin (includes one patient treated with quinidine 500 mg b.d. for one cycle)

| *No. patients* | *Outcome* |
|---|---|
| 6 | completed planned 8 cycles |
| 7 | off no response/PD |
| 2 | off quinidine toxicity<br>1 cinchonism, 1 dizziness |
| 1 | off myelosuppression, 6 cycles |
| 1 | off skin reaction, 6 cycles |
| 1 | off nausea and vomiting, 7 cycles |
| 1 | off oral toxicity after 1 cycle |
| 1 | off declined last cycle with PR |
| 1 | off because crushing durules |

**Table II** Summary of haematological toxicity for treatment cycles with and without quinidine

| | *Cycle 1, without quinidine* | *cycles 2–4, with quinidine* | *Cycles 4–8, with quinidine* |
|---|---|---|---|
| Median WBC nadir ($\times 10^9\ l^{-1}$) | 2.3 | 1.5 | 1.6 |
| Range | 1.1–4.5 | 1.0–5.9 | 0.7–5.2 |
| Median platelet nadir ($\times 10^9\ l^{-1}$) | 175 | 158 | 156 |
| Range | 85–565 | 37–565 | 54–307 |

**Table III** Summary of patient plasma quinidine levels achieved with different quinidine doses

| *Number of patients* | *Quinidine dose* | *Number of cycles* | *Median quinidine level (μmol $l^{-1}$)* |
|---|---|---|---|
| 3 | 1 g b.d. | 1 | 12 |
| 8 | 500 mg b.d. | 28 | 7.4<br>(2.9–13.2) |
| 20 | 250 mg b.d. | 77 | 5.6<br>(2.1–22.1) |

In summary, treatment of patients with advanced breast cancer with a combination of quinidine and epirubicin appears feasible. The combination does not appear to produce more toxicity than epirubicin alone, and at a dose of 250 mg b.d. quinidine levels equivalent to those active *in vitro* are achievable in patients.

The question remains as to whether an improvement in response rate and survival can be achieved in the clinic, by the use of quinidine to overcome anthracycline resistance. In view of our data on MDR expression in samples from previously untreated patients, we have chosen to address this question in a randomised, prospective, placebo-controlled trial, using epirubicin as initial chemotherapy for patients with advanced disease.

We would like to acknowledge support from the Cancer Research Campaign.

### References

BROWN, A., KEITH, N., STALLARD, S. & KAYE, S.B. (1989). Expression of *mdr*1 and *gst*-pi in breast tumours: correlations with chemoresponsiveness *in vitro*. *Proc. Am. Assoc. Cancer Res.*, **30**, 516.

CARTER, S.K. (1976). Adriamycin–a review. *J. Natl Cancer Inst.*, **55**, 1265.

KAYE, S.B. (1988). The multidrug resistance phenotype. *Br. J. Cancer*, **58**, 691.

STALLARD, S. & KAYE, S.B. (1989). Reversal of resistance in the breast cancer cell line MCF-7/Adr$^R$ was most effective with the modulating agent quinidine. *Br. J. Cancer*, **60**, 500.

TSURUO, T., HDA, H., KITANI, Y., YOKATA, K., TSUKAGOSHI, S. & SAKURAI, Y. (1984). Effects of quinidine and related compounds on cytoxicity and cellular accumulation of vincristine and adriamycin in drug resistant tumour cells. *Cancer Res.*, **44**, 4303.

YUSA, K. & TSURUO, T. (1989). Reversal of multidrug resistance of verapamil: direct binding of verapamil to P-glycoprotein on specific sites and transport of verapamil outward across the plasma membrane of K562/ADM cells. *Cancer Res.*, **49**, 5002.